# Hepatitis D virus infection in a large cohort of immigrants in southern Italy: a multicenter, prospective study

**DOI:** 10.1007/s15010-022-01938-0

**Published:** 2022-10-12

**Authors:** Mariantonietta Pisaturo, Loredana Alessio, Alessandra Di Fraia, Margherita Macera, Carmine Minichini, Emanuele Cordua, Lorenzo Onorato, Gaetano Scotto, Giovanni Di Caprio, Federica Calò, Caterina Sagnelli, Nicola Coppola

**Affiliations:** 1 Department of Mental Health and Public Medicine, Section of Infectious Diseases, Second University of Naples, University of Campania Luigi Vanvitelli, Via: L. Armanni 5, 80131 Naples, Italy; 2 Medical Center, Centro Sociale ex Canapificio, Caserta, Italy; 3 Medical Center, Centro di Accoglienza “La Tenda di Abramo”, Caserta, Italy; 4 Medical Center, Centro per la Tutela Della Salute Degli Immigrati, Naples, Italy; 5 Infectious Diseases Unit, AORN Sant’Anna e San Sebastiano, Caserta, Italy; 6 Medical Center, Centro Suore Missionarie Della Carità, Naples, Italy; 7 Medical Center, Centro Borgoroma, Foggia, Italy; 8 Infectious Diseases Unit, Foggia, Italy

**Keywords:** HDV infection, Anti-Delta, HBV infection, Immigrants, Viral hepatitis

## Abstract

**Background:**

Since few data are available in the literature on the prevalence of anti-Delta-positive subjects in immigrant populations, the aim of the present study was to evaluate the demographic and virological characteristics of HDV infection in a large cohort of immigrants living in southern Italy.

**Methods:**

Between January 2012 and February 2020 all immigrants attending one of the 5 first- level centers were enrolled and screened for HBsAg, the HBsAg-positive for anti-Delta and if positive, for HDV-RNA and HDV genotype.

**Results:**

Of the 3521 immigrants observed in the study period, 3417 (97.0%) agreed to be screened; they were mainly males (61%), with a median age of 27 years (IQR 8–74) and came prevalently (58%) from sub-Saharan Africa.

Of the 3417 patients enrolled, 319 (9%) subjects were HBsAg-positive, and of those, 8 (2.5%) were anti-Delta-positive. No difference in the demographic and epidemiological characteristics was observed between the anti-Delta-negative vs -positive. Of the 8 anti-Delta-positive subjects, only one was HDV-RNA-positive (viral load: 7050 IU/mL), genotype 1, with clinical signs of cirrhosis.

**Conclusions:**

the present study showed a prevalence of HDV of 2.5% in a large cohort of asymptomatic immigrants, suggesting the need for screening campaigns for viral infections including delta hepatitis in this population.

**Supplementary Information:**

The online version contains supplementary material available at 10.1007/s15010-022-01938-0.

## Introduction

Hepatitis D virus (HDV) is the smallest known human virus, characterized by a peculiar morphologic aspect and by a replication cycle depending on the presence of hepatitis B virus (HBV) infection. In association with HBV, HDV induces acute and chronic liver lesions, frequently severe, and, at times, life‐threatening [1–4]. In fact, once chronic HDV infection is established, the pre‐existing chronic liver disease caused by HBV usually worsens [5] and shows a more frequent and more rapid progression to cirrhosis [6, 7] and liver decompensation than that observed in patients with chronic hepatitis by HBV mono-infection [8, 9].

HDV infection is spread all over the world, with different frequencies in different geographical areas. Globally, it is estimated that 5% of HBsAg carriers are also infected with HDV, suggesting that 8 to 20 million people are chronic carriers of HBV/HDV coinfection [10–18]. In Italy the prevalence of HDV infection in HBsAg-positive subjects ranges from 9.7 to 12%, with evidence of an increasing prevalence of HDV infection in the immigrant population in recent years [19–25]. In fact, the prevalence of HBV and HDV infections is high in geographical areas that are the areas of origin of the majority of immigrants arriving in Italy and in Western countries. For example, HDV is highly endemic in several African countries and in some countries in South America, the Middle East, central and northern Asia, and Eastern Europe [11]. A systematic review of the prevalence of HDV infection in HBsAg‐positive populations in sub‐Saharan Africa showed a pooled seroprevalence in western Africa in HBsAg-positive general populations and in patients with liver disease of 7.33% and 9.57%, respectively, of 25.6% and 37.8% in central, and 0.05% in the general populations of eastern and southern Africa [12]. Another study performed in northern African countries showed an overall prevalence of HDV infection in HBsAg chronic carriers of 5% in the general populations and of 20.7% in patients with liver disease [13].

However, few data have been published so far on the prevalence of HDV infection in immigrant populations in Western countries and even poorer is the information on the virological and clinical aspects of HDV infection in this population.

To help improve the knowledge on this topic, we designed a multi-center prospective study to evaluate the demographic and virological characteristics of HDV infection in a large cohort of immigrants living in southern Italy.

## Patients and methods

### Study protocol and setting

Between January 2012 and February 2020 a prospective, multicenter, collaborative study, based on the long-term active cooperation between three 3rd level units of Infectious Diseases and five 1st level clinical centers was designed [26–29]. The present study is based on a screening program that began in January 2012 and ongoing until the COVID 19 pandemic in Italy in February 2020, in the 5 first-level centers participating in the study, 2 in Naples, 2 in Caserta and 1 in Foggia. These three cities give hospitality to a large immigrant population from Africa, middle and eastern Asia and eastern Europe. These five first-level clinical centers are general practice clinics attended mainly by low-income refugees and undocumented immigrants for low back pain, headache, itching, cough, hypertension and allergic symptoms; thus, they have proven experience in managing vulnerable groups and are greatly appreciated by the immigrants.

By the term “immigrant” we refer to a heterogeneous population including undocumented immigrants, asylum seekers (people fleeing persecution or conflicts seeking international protection under the 1951 Refugee Convention on the Status of refugees), refugees (asylum seekers whose request has been approved) and economic immigrants (people whose primary motivation for fleeing their home country is economic gain). We have included both newly arrived immigrants and subjects who have been living in Italy for a longer time.

All immigrants consecutively assessed for a clinical consultation at one of the five centers from January 2012 to February 2020 were enrolled. During clinical consultations, asked for by the patient, a physician and a cultural mediator explained the importance of being tested for the principal parenteral viral infections and offered testing free of charge, in anonymity (recording only the center number and patient’s number), in full accordance with the Italian privacy law regarding observational studies. Immigrants who agreed to undergo screening had to sign a screening adhesion and signed informed consent, written in the immigrant's language. The information was collected from each participant through a questionnaire administered by the research investigators with the assistance of a cultural mediator whose presence provided security and protection.

The questionnaire recorded age, sex, geographical origin, date (month and year) of immigration, level of education, religion, family history, cohabitation details, sexual orientation and practices including condom use, history of hepatitis B virus (HBV) vaccination, surgery, dental care, tattooing, body piercing, use of drugs, blood transfusion, tribal rituals, abortion and information on previously documented personal and family infections of HBV, HDV, hepatitis C virus (HCV) and human immunodeficiency virus (HIV).

The data relating to the epidemiological characteristics were collected in an electronic database.

All subjects included in the study were screened for hepatitis B surface antigen (HBsAg), anti-HCV and anti-HIV; the HBsAg-positive were screened also for anti-Delta. None of the patients enrolled was aware of their serological status. In anti-HDV-positive subjects HDV-RNA was sought and HDV genotype identified.

Participants who were positive for HBsAg and/or anti-Delta, and/or anti-HCV-positive and/or anti-HIV positive were referred to one of the two tertiary units of infectious diseases operating in the same city and involved in the study for further investigation, monitoring and possible treatment.

HBsAg positivity was considered an index of ongoing HBV infection and HBsAg negativity/anti-HBc positivity status as a marker of a previous HBV infection. Anti-HCV positivity was considered an indication of past or ongoing HCV infection and anti-HIV positivity as a marker of ongoing HIV infection. Anti-Delta positivity was considered an indication of past or ongoing HDV infection.

Liver cirrhosis was diagnosed on the basis of a liver biopsy showing a fibrosis score of F4 according to the METAVIR score, or F5 or F6 according to Ishak, or a Fibroscan score of more than 12.4 kPa or on the basis of the presence of unequivocal clinical signs including a blood platelet count lower than 100,000/mm^3^, presence of ascites, porto-systemic encephalopathy, esophageal varices and ultrasound evidence characterizing liver cirrhosis.

The study was approved by the Ethics Committee of the Azienda Ospedaliera Universitaria of the University of Campania Luigi Vanvitelli (214/2012; 481/2018).

### Testing procedure

Serum samples were tested for HBsAg, total anti-HCV, anti-HIV, anti-HBc and hepatitis B surface antibodies (anti-HBs) by commercial immunoenzymatic assays (Abbott Laboratories, North Chicago, IL, United States: AxSYM HBsAg (V.2) M/S for HBsAg, AXSYM HCV 3.0 for anti-HCV, AXSYM HIV 0.5 COMBO for anti-HIV, AXSYM core for anti-HBc and AXSYM AUSAB for anti-HBs). Anti-HIV reactivity was always confirmed by a Western blot assay (Genelabs Diagnostics, Science Park Drive, Singapore), which identifies both HIV-1 and HIV-2 strains. Anti-delta was performed in HBsAg-positive subjects by HDV Ab Elisa (Dia.pro diagnostic bioprobes, Sesto San Giovanni, Milano, Italy).

Circulating HBV DNA was quantified by real-time polymerase chain reaction (PCR) in a Light cycler 1.5 (Roche Diagnostics, Branchburg, NJ, United States) [30]; the HBV genotype was determined in HBV-DNA-positive samples, as previously described [31]. LLoQ for the HBV DNA test was 10 UI/ml.

Serum HDV-RNA was quantified by a commercial HDV Quantification-Detection Kit (Anatolia Geneworks BOSPHORE, İstanbul, Turkey). LLoQ for the HDV RNA test was 45 copie/ml.

For HDV genotype, after extraction using a commercially available kit (QIAmp RNA blood mini-kit, Qiagen Inc., USA), RNA was amplified with HotStarTaq Polymerase (Qiagen Inc., USA) using the following primer pairs HDV_F1-50CTTAGCCATCCGAGTGGACG and HDV_R1-50GTCCAGCAGTCTCCTCTTTACA for the first PCR and HDV_F2-50AGACGCAAACCTGYGAGTGG and HDV_R1 (mentioned before) for the second PCR. PCR-products were purified and sequenced by using different overlapping sequence-specific primers and a BigDye terminator v. 3.1 cycle sequencing kit (Applied-Biosystems, Foster City, CA, USA). The sequences were analyzed using SeqScape-v.2.5 software (Thermo Fisher Scientific, Waltham, MA, USA). HDV genotypes were assessed by phylogenetic analysis using HDV sequences obtained from population-based sequencing. Nucleotide sequences of HDV were compared to reference sequences representing all known HDV genotypes retrieved from the Genbank (HDV accession numbers: AB51639, FJ709464, AY090459, DQ899146, DQ899142, AB036920, AF223965, GU563556, FN594748, JN664942, EU594434, GU456636, JN182318, GQ331047, AB194951, AY934764, FJ692613, FJ023659, FJ023664, AB644280, AB554025, AB644286, AB554019, AB644287, AB540583, AP011106, HM011493, AB644284, EU410080, EU670263, GU721029, AP011108, GQ358158, AB697490, DQ089801; HDV accession number: X04451, X60193, L22063, AF018077, AJ584848, AJ584847, AJ584844, AJ584849) using MEGA 5.02 (https://www.megasoftware.net/).

### Statistical analysis

Continuous variables were summarized as mean and standard deviation, and categorical variables as absolute and relative frequencies. Differences in mean values were evaluated by Student’s t-test and the chi-squared test was applied to categorical variables. A *p* value < 0.05 was considered to be statistically significant.

## Results

In the study period we observed 3521 immigrants in the five first-level clinical centers. In particular, 3417 (97.0%) agreed to be screened and were enrolled in the present study (Fig. 1 in supplementary data). Table 1 shows the characteristics of the subjects enrolled. They were mainly males (61%), with a median age of 27 years (IQR 8–74). The average number of months spent in Italy was 28.3 (standard deviation, SD ± 45.1). As regards the geographical area of origin, 2066 subjects (58%) came from sub-Saharan Africa, 642 (19%) from Asia, 310 (9%) from Eastern Europe, 141 (4%) from northern Africa, 34 (0.42%) from South America; for 224 (1%) the geographical origin was not known. Three hundred and nineteen subjects were HBsAg-positive (9%), 53 anti-HIV positive (1.5%), 101 anti-HCV-positive (3%) and 1,332 were HBsAg-negative but anti-HBc-positive (39%). Table A in supplementary data described characteristics of HBsAg-negative subjects versus HBsAg-positives ones.

**Fig. 1:**
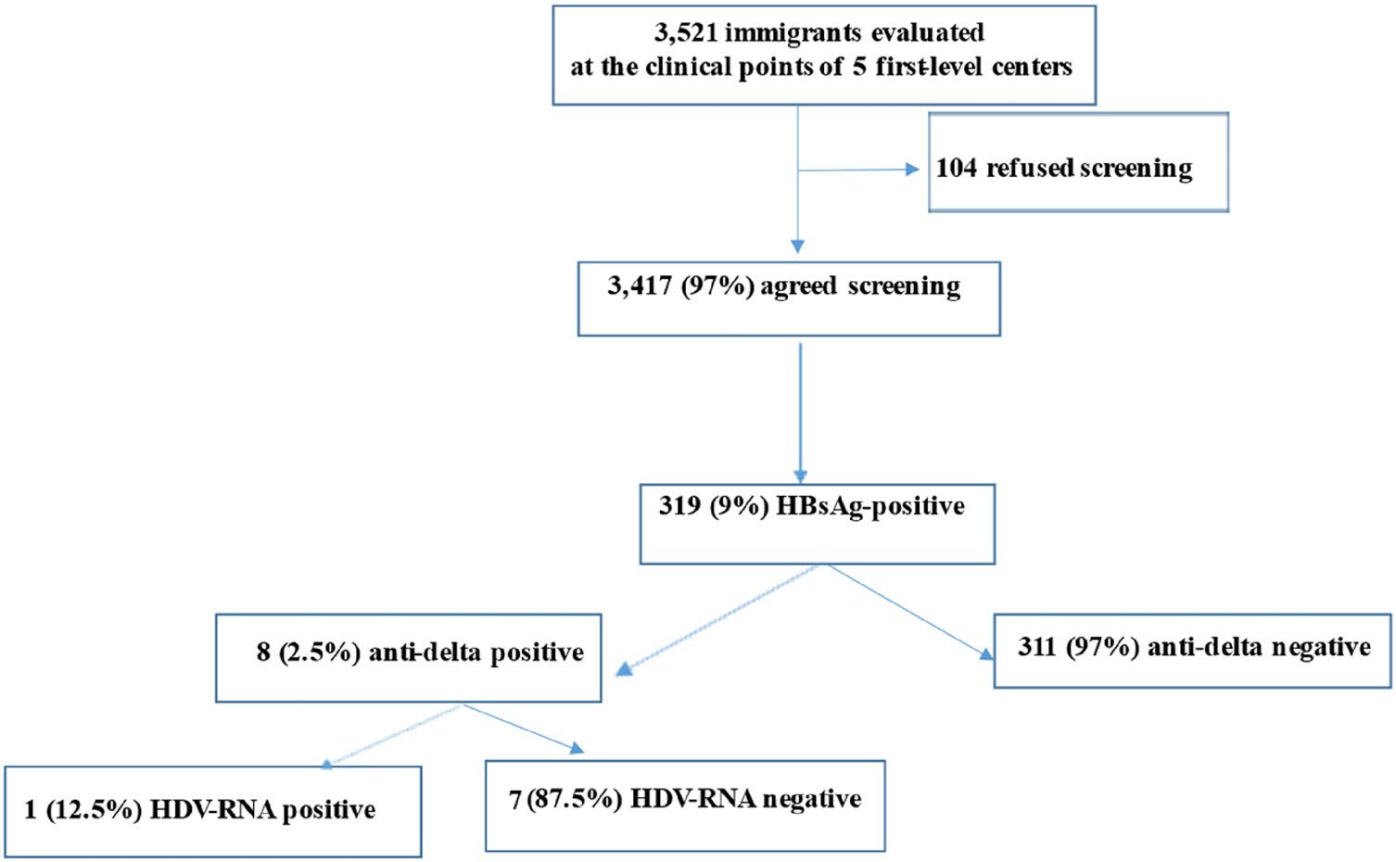
Flow-chart of the enrolled patients

**Table 1:** Demographic and serological characteristics of the 3417 enrolled immigrants

Number of patients	3417
Age, median (IQR)	27 (8–74)
Males, *n* ° (%)	2805 (61)
Months of stay in Italy, media (SD)	28.3 (± 45.1)
Geographical area of origin, *n* ° (%)	
Eastern Europe	310 (8.89)
Asia	642 (18.7)
North Africa	141 (4.12)
Sub-Saharan Africa	2066 (57.8)
South America	34(0.43)
Not known	224 (1.05)
Serum status, *n* ° (%)	
HBsAg positive/anti-HCV negative/anti-HIV negative	300 (9)
HBsAg positive/anti-HIV positive/anti-HCV negative	8 (0.2)
HBsAg positive/Anti-HCV positive/anti-HIV negative	9 (0.2)
HBsAg positive/anti-HIV positive/Anti-HCV positive	2 (0.05)
HbsAg-negative/Anti-HBc positive	1332 (39)
HBsAg negative/ Anti-HCV positive/anti-HIVnegative	101 (3)
HBsAg negative /Anti-HCV positive/anti-HIV positive	6 (0.1)
HBsAg negative/ Anti- HIV positive/anti-HCV negative	60 (1.8)
HBsAg negative/anti-HCV negative/anti-HIV negative/anti-HBc negative	1606 (47)

The 319 HBsAg-positive subjects were analyzed. They were mainly males (90%) and young [median age 28 years (IQR 21)] and with a mean stay in Italy of 23.8 months (SD ± 44). Eight (2.5%) were HIV coinfected, 9 (2.8%) HCV coinfected, 2 (0.6%) HIV and HCV coinfected. Of the 319 HBsAg-positive subjects, 8 (2.5%) were anti-HDV-positive (Fig. 1, Table A in supplementary data).

Table 2 compares the epidemiological and virological characteristics of the 319 HBsAg-positive patients based on serum HDV status. There was no difference in gender prevalence between the anti-Delta-negative vs -positive subjects (93% vs 87.5%; *p* = 0.42), nor in median age [26.5 (IQR 32–21) vs 21.5 (IQR 30.5–19.25); *p* = 0.18], nor in the area of origin: there was a higher prevalence of subjects from sub-Saharan Africa in both groups (86% vs. 100%). The average of the months of stay in Italy was higher in anti-HDV-positive subjects (22 ± 37.7 vs. 7 ± 8.2 months), but with no significance to the statistical analysis (*p* = 0.2). The most frequent risk factors were unprotected sexual intercourse and intramuscular therapy, (70% vs. 50%, *p* = 0.20; and 82% vs. 62.5%, *p* = 0.01, respectively).

**Table 2:** Epidemiological and virological characteristics of the 243 HBsAg-positive subjects based on serum anti-HDV status

	Anti-Delta negative	Anti-Delta positive	*p* value
Number of patients	311	8	
Age, median (IQR)	26.5 (32–21)	21.5 (30.5—19.25)	0.18
Males, *n* ° (%)	289 (93)	7 (87.5)	0.42
Geographical area of origin, *n* ° (%)			
Eastern Europe	20 (6.4)	0	//
Asia	19 (6)	0	//
Northern Africa	3 (0.9)	0	//
sub-Saharan Africa	268 (86)	8 (100)	//
South America	1 (0.432)	0	//
Months spent in Italy, media (+ SD)	22 (± 37.7)	7 (± 8.2)	0.23
Years of schooling, media (+ SD)	4.4 (± 3.9)	5.3 (± 3.55)	0.59
Religion, *n* ° (%)			
Muslims	226 (72.6)	6 (75)	0.54
Christians	58 (18)	2 (25)	0.55
Catholics	5 (1.6)	0	//
Orthodox	12 (3.8)	0	//
Other religion	10 (3.2)	0	//
Housing conditions			
Stable apartment, *n* ° (%)	254 (82)	6 (75)	0.51
Roommates, media (+ SD)	4.5 (± 5.1)	3.5 (± 2.3)	0.62
Bathrooms, average (+ SD)	1.3 (± 1.1)	1.1 (± 0.4)	0.71
Consumption of alcohol, *n*° (%)	19 (8)	0	0.42
Risk factors, *n* ° (%)			
Drug addiction	0	0	
Sexual intercourse without a condom	158/223 (70)	4 (50)	0.20
Surgical interventions	45/231 (19.4)	0	//
Dental procedures	81/227 (36)	1 (12.5)	0.17
Intramuscular therapy	186/227 (82)	5 (62.5)	0.01
Tattoo	13/231 (6)	0	//
Pearcing	16/231(7)	0	//
Tribal scars	41/271 (15)	1 (12.5)	0.83
Sexual orientation			
Heterosexuals, *n* ° (%)	231 (74)	8 (100)	//
Stable partner, *n* ° (%)	100 (32)	4 (50)	0.28
Serum status, *n* ° (%)			
HBsAg positive/anti-HIV positive/anti-HCV negative	8 (2.5)	0	//
HBsAg positive/Anti-HCV positive/anti-HIV negative	9 (2.8)	0	//
HBsAg positive/anti-HIV positive/ Anti-HCV positive	2 (0.6)	0	//
HBV-DNA positivity, *n*° (%)	143/235 (61)	3 (37)	0.18
HBV-DNA, IU/mL (median, IQR)	1310 (0–7.71 × 109)	273 (0–2379)	0.82
HBV genotypes, (*n*, %)			
A	15/235 (6.4)	not identifiable^	
C	3/235 (1.3)		
D	11/235 (4.7)		
E	61/235 (25.9)		
Not known	65/235 (27.7)		
Not genotyped due to low viral load	80/235 (34)		
HDV-RNA positivity, *N* (%)	Not applicable	1^^	

LLoQ for the HBV DNA test was 10 UI/ml

LLoQ for the HDV RNA test was 45 copie/ml

^Not identifiable because HBV-DNA-negative or at low viral load ^^HDV genotype 1

HBV DNA was detectable in 143 (61%) of HBsAg-positive/anti-Delta-negative subjects (data available for 235 subjects) and in 3 (37%) of the 8 HBsAg and anti-Delta-positive (*p* = 0.18); HBV load was similar in the two groups (Table 2). As regards the HBV genotype, identified only in HBV DNA positive subjects, genotype E was the most prevalent, then genotype D and A, and finally genotype C (Table 2). In none of the anti-Delta-positive subjects was the HBV genotype identified because they were HBV DNA-negative or at a low viral load. All anti-Delta-positive subjects were evaluated for serum HDV-RNA and HDV genotype. Only one patient resulted positive for HDV-RNA, genotype 1. This patient was a 46-year-old, had HDV viremia of 7050 IU/mL and an ultrasound diagnosis of compensated cirrhosis. In May 2016 he started therapy with pegylated interferon, interrupted in November 2016 because of thrombocytopenia and started therapy with entecavir 1 mg/die.

## Discussion

In the present prospective multicenter study, we evaluated the HDV prevalence in a large cohort of 3,417 immigrants in a screening program, free of charge and of bureaucratic procedures, at centers they had been consulting for clinical, social or legal problems. The evaluation of the prevalence of HDV infection in immigrant populations is important as since 2011, the European Union (EU) has faced one of the greatest influxes of immigrants ever occurring. Moreover, because of its geographical position in the center of the Mediterranean Sea, Italy has been greatly involved in immigration from Africa, in particular from sub-Saharan Africa, and also from Asia and Eastern Europe. The present cohort of immigrants can be considered representative of this migratory flow, since more than half of the immigrants were born in sub-Saharan Africa.

The acceptance rate of our screening program was high (97.0%), probably due to the experience of the physicians and cultural mediators of our first-level medical centers in the management of vulnerable, difficult-to-reach groups, such as undocumented immigrants and low-income refugees emarginated by language, cultural and social barriers, and also the rapid referral to Infectious Disease Units in the same territory for further investigation and treatment.

The prevalence of HBV infection in our cohort was of 9%; of these 319 HBsAg-positive immigrants, whose serological status was unknown before the present screening program, 8 (2.5%) resulted anti-HDV-positive. They were prevalently males, young and from sub-Saharan Africa, with no specific parenteral risk factor.

The data from the present study is interesting, since few data exploring HDV infection in immigrants are available in the literature and show different HDV prevalence’s in different countries and in different studies ranging from less than 1% to about 43% [25]. However, the data available are from populations often of low sample size and prevalently from the hospital setting. In fact, the majority of studies evaluated the prevalence of HDV infection by enrolling HBsAg-positive subjects admitted to hospital clinical centers, distinguishing between natives and immigrants. For example, in a multicenter cross-sectional study performed in the UK, of 55 HDV-infected patients identified, 50 (91%) were immigrants and 27 (49%) had evidence of cirrhosis [32].

However, in several Western countries there has been an increasing prevalence of HDV infection in immigrant populations in recent years. A study conducted by Ordieres et al*.* in Spain [33] described epidemiological changes of HDV infection in a retrospective study on 1,215 HBsAg-positive patients enrolled over 30 years and grouped according to the year they had been observed in 6 interval-groups of 5 years each. Among the 1,064 patients born in Spain the highest prevalence (13%) was reached in the first interval-group (1983–1987) and the lowest (4%) in the last two interval-groups (from 2003 to 2012); conversely, among the 151 immigrants included, the highest prevalence (10.7%) was reached in the last interval-group (2008–2012). Similar results were reported in a study conducted in Greece [34], which showed an overall stable prevalence of HDV infection in 2,137 HBsAg carriers, 4.1% in the interval-group from 1997 to 2003 and 4.4% from 2004 to 2010, with a significant difference, however, between Greek (2.8%) and immigrant patients (7.8%), most of whom from Balkan countries. As regards Italy, in 2009 a multicenter study [35] on 1386 HBsAg-positive patients, 104 of whom were from Eastern Europe or Asia, found no difference in the HDV prevalence between Italians (8%) and immigrants (8.3%). Three years later, a national survey involving 74 Italian liver units [36] showed a 5.5% prevalence of HDV infection in 730 HBsAg-positive patients from Eastern Europe, eastern Asia or sub-Saharan Africa, and of 8.2% in 2575 Italian HBsAg-positive patients. In the same year, another study conducted on 488 HBsAg-positive subjects [37] including 107 immigrants, found no difference in the HDV prevalence between Italian and immigrant subjects (7% and 7.3%, respectively). Instead, more recently, evaluating HDV prevalence in 786 HBsAg-positive subjects in 9 liver units prospectively enrolled for a 6-month period in 2019, the anti-HDV overall prevalence was 9.9%, 6.4% in Italian natives and 26.4% in non-natives [38].

However, all these and other data on this topic come from studies performed in liver units and, thus, the impact of HDV infection in the general immigrant population is, today, still unknown. Thus, the data of the present study may be useful to clarify the prevalence of HDV in the general population of immigrants and a warning for the Italian Healthcare Authorities to develop suitable cost-effective screening policies in this setting.

Moreover, few are also the data from the literature on the demographic, clinical and virological characteristics of immigrants with HDV infection. According to our data, subjects with HBV/HDV coinfection were prevalently young men from sub-Saharan Africa. However, no difference in age, country of origin or risk factor for parenteral infection was observed in HBsAg-positive subjects between the anti-delta-positive and -negative, suggesting the need for HDV screening in all HBsAg-positive subjects.

In conclusion, the present study showed a prevalence of HDV of 2.5% in a large cohort of immigrants enrolled in first-level units without signs and symptoms of liver disease. No epidemiological differences were observed in HBsAg-positive subjects according to HDV serology, and the HDV-RNA prevalence is low. Since the high prevalence of HBV infection in immigrants and the severe presentation of HDV infection in the case of viral replication, we believe it is appropriate for the scientific communities to work to carry out screening campaigns for viral infections including delta hepatitis in immigrant populations and to work to ensure the best possible treatment for all.

## Supplementary Information

Below is the link to the electronic supplementary material.

Supplementary file1 (DOCX 21 KB)

## Data Availability

The data may be required to the corresponding author, Prof Nicola Coppola (Nicola.coppola@unicampania.it).
