# Correction: Hepatitis D virus infection in a large cohort of immigrants in southern Italy: a multicenter, prospective study

**DOI:** 10.1007/s15010-023-02002-1

**Published:** 2023-04-06

**Authors:** Mariantonietta Pisaturo, Loredana Alessio, Alessandra Di Fraia, Margherita Macera, Carmine Minichini, Emanuele Cordua, Lorenzo Onorato, Gaetano Scotto, Giovanni Di Caprio, Federica Calò, Caterina Sagnelli, Nicola Coppola

**Affiliations:** 1 Department of Mental Health and Public Medicine, Section of Infectious Diseases, Second University of Naples, University of Campania Luigi Vanvitelli, Via: L. Armanni 5, 80131 Naples, Italy; 2 Medical Center, Centro Sociale ex Canapificio, Caserta, Italy; 3 Medical Center, Centro di Accoglienza “La Tenda di Abramo”, Caserta, Italy; 4 Medical Center, Centro per la Tutela Della Salute Degli Immigrati, Naples, Italy; 5 Infectious Diseases Unit, AORN Sant’Anna e San Sebastiano, Caserta, Italy; 6 Medical Center, Centro Suore Missionarie Della Carità, Naples, Italy; 7 Medical Center, Centro Borgoroma, Foggia, Italy; 8 Infectious Diseases Unit, Foggia, Italy

**Correction: Infection (2022) 50:1565–1572** 10.1007/s15010-022-01938-0

The original version of this article unfortunately contained a mistake. In the author list, the first and last names were incorrectly structured. The corrected author list is given above.

Further in Fig. 1 of this article there was an error: 7 (not 1) (87.5%) were HDV-RNA negative; the figure (Fig. 1) should have appeared as shown below.

**Fig. 1:**
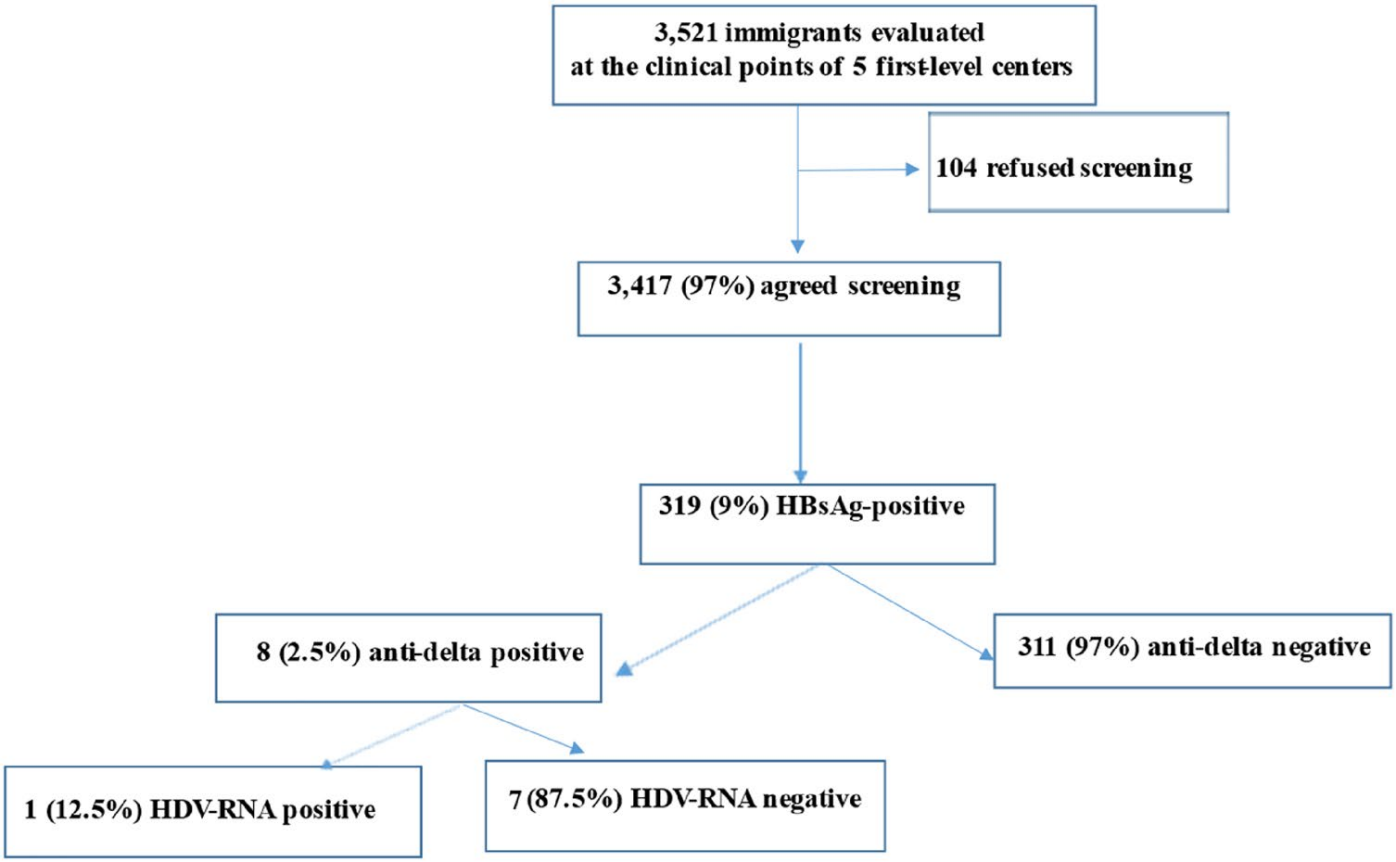
Flow-chart of the enrolled patients

The original article has been corrected.

