# Supplementary material for: Hepatitis D virus infection in a large cohort of immigrants in southern Italy: a multicenter, prospective study
Source: Infection. 2022 Oct 12;50(6):1565–72. doi: 10.1007/s15010-022-01938-0 (PMC9554856; doi:10.1007/s15010-022-01938-0)
Supplement: Supplementary file 1 — Supplementary file1 (DOCX 21 KB) [file 15010_2022_1938_MOESM1_ESM.docx]

**Table A. Epidemiological and virological characteristics of the HBsAg-negative subjects versus the HBsAg-positives ones**

|  | **HBsAg negative subjects** | **HBsAg positive** **subjects** | **P value** |
| --- | --- | --- | --- |
| **Number of patients** | 3098 | 319 |  |
| **Age, median (IQR)** | 23 (22) | 28 (21) | 0.32 |
| **Males, n ° (%)** | 2615 (84) | 289 (90) | 0.03 |
| **Geographical area of origin n ° (%)**  **Eastern Europe**  **Asia**  **Northern Africa**  **sub-Saharan Africa**  **South America**  **Not known** | 298 (9.6)  634 (20)  138 (4)  2003 (64)  20 (0.6)  81 (3) | 10 (7)  18 (6)  3 (1)  255 (80)  1 (0.3)  0 | < 0.00  < 0.00  0.01  < 0.00  0.67 |
| **Months spent in Italy, media (+SD)** | 27,6+39 | 23,8+44 | 0.13 |
| **Years of schooling, media (+ SD)** | 6,3+4,7 | 4,7+4,1 | 0.000 |
| **Religion n ° (%)**  **Muslims**  **Christians**  **Catholics**  **Orthodox**  **Buddist**  **other religion**  **not known** | 1995 (64)  830 (27)  88 (3)  195 (6)  11 (0.3)  18 (0.6)  37 (1.1) | 226 (71)  58 (18)  5 (1.5)  12 (4)  0  3(1)  13 (4) | 0.02  0.00  0.18  0.71  //  0.43  0.00 |
| **Housing conditions**  **Stable apartment, n ° (%)**  **Roommates, media (+SD)**  **Bathrooms, media (+SD)** | 2625 (84)  4,6+6,1  1,3+1,3 | 254 (80)  4,5+5,1  1,3+1,1 | 0.017  0.71  0.81 |
| **Consumption of alcohol, n°(%)**  **Not known** | 2006 (65)  93 (3) | 39 (12)  123 (39) | < 0.00  < 0.00 |
| **Risk factors, n ° (%)**  **Drug addiction**  **Sexual intercourse without a condom**  **Surgical interventions**  **Dental procedures**  **Intramuscular therapy**  **Tattoo**  **Pearcing**  **Tribal scars** | 15/2339 (0.6)  1418/2237 (63)  534/2474 (21)  768/2438 (31)  1876/2363 (79)  185/2472 (7)  192/2462 (8)  331/855 (39) | 0  158/231(68)  45/239 (19)  81/235 (34)  189/225 (84) 13/239 (5.4)  16/239 (6.6)  41/88 (46.5) | 0.46  0.32  0.35  0.09  0.24  0.54  0.07 |
| **Sexual orientation**  **Heterosexuals, n ° (%)**  **Stable partner, n ° (%)** | 2319 (75)  1057 (34) | 231 (72)  100 (31) | 0.34  0.31 |
| **Serum status for HCV and HIV, n ° (%)**  **anti-HCV positive**  **anti-HIV positive**  **anti-HIV positive/ Anti-HCV positive** | 101 (3.2)  60 (2)  6 (0.1) | 9 (2.8)  8 (2.5)  2 (0.6) | 0.85  0.15  0.12 |
| **Serum status for HDV, n° (%)** | Not applicable | 8 (2.5) | Not applicable |
